# Disposable Airway Pressure Manometers for Endotracheal Tube Cuff Inflation

**DOI:** 10.3390/ani13030475

**Published:** 2023-01-30

**Authors:** Moriz Ettore Klonner, Giorgio Mattaliano, Vincenzo Casoria, Claus Vogl, Christina Braun

**Affiliations:** 1 Clinical Unit for Anaesthesiology and Perioperative Intensive Care Medicine, Vetmeduni Vienna, 1210 Vienna, Austria; 2 Southern County Veterinary Specialists, Ringwood BH4 3JW, UK; 3 Unit of Molecular Genetic, Institute of Animal Breeding and Genetics, Vetmeduni Vienna, 1210 Vienna, Austria

**Keywords:** cuff pressure, endotracheal tube, high-volume, low-pressure, manometer

## Abstract

**Simple Summary:**

Evidence suggests that the cuff pressure of endotracheal tubes with high-volume, low-pressure cuffs should be maintained between 20 and 30 cmH_2_O. Blind techniques for cuff inflation have been shown to rarely result in an optimal cuff pressure, even when used by experienced anaesthesiologists. The authors therefore emphasise the necessity for direct measurement of the cuff pressure using a manometer. Despite a wide array of commercially available cuff pressure manometers, many veterinary facilities still use blind techniques or repurposed aneroid blood pressure manometers, which have been proven unreliable for this application. With cost being a major concern, especially for smaller veterinary facilities, the aim of this study was to find a cost-effective alternative to commercially available cuff manometers and test them for their usability, accuracy, precision and repeatability. This study assessed the performance of two disposable airway manometers for endotracheal tube cuff inflation in a benchtop model. Both of the tested devices present cost-effective and accurate alternatives to commercial cuff manometers.

**Abstract:**

This study aimed to assess the performance, accuracy, precision and repeatability of two single-use airway pressure manometers as a cost-effective alternative for inflation of endotracheal tubes with high-volume, low-pressure cuffs. The manometers were tested in a bench top model against a U-tube manometer. Eighteen units of each device were tested. Three consecutive measurements were performed at pressures of 20, 25 and 30 cmH_2_O each. The mean ± SD of the recorded pressures and maximum deviation from the target pressures were calculated for each device and each target pressure. For device A, the mean ± SD pressures were 19.6 ± 0.7, 23.6 ± 0.8 and 28.3 ± 0.8 cmH_2_O; for device B, the mean ± SD pressures were 19.3 ± 0.6, 24.3 ± 0.9 and 29.2 ± 0.67 cmH_2_O for target pressures of 20, 25 and 30 cmH_2_O, respectively. The bias for device A was −0.4, −1.4, and −1.7 cmH_2_O and for device B, −0.7, −0.7, and −0.8 cmH_2_O for target pressures of 20, 25, and 30 cmH_2_O, respectively. Both devices showed results comparable to those reported for commercial cuff manometers. They represent inexpensive tools that provide clinically sufficient accuracy, precision and repeatability for ETT cuff inflation between pressures of 20 and 30 cmH_2_O.

## 1. Introduction

Airway complications related to excessive endotracheal tube cuff pressure have been reported as early as the late 1960s [1,2,3]. Since then, evidence has arisen suggesting that the intra-cuff pressure of endotracheal tubes (ETT) with high-volume, low-pressure (HVLP) cuffs should be kept between 20 and 30 cmH_2_O to minimize intubation-related complications in humans [4,5,6,7]. Although no clear consensus has been reached in veterinary medicine, and results have mainly been extrapolated from human literature, the same cuff pressure range is generally recommended in animals [8,9]. Too low an intra-cuff pressure can lead to fluid leakage and bacterial transmigration into the lower airway, while too high an intra-cuff pressure can cause ischemic damage to the tracheal mucosa [4,10,11]. Complications ranging from coughing, sore throat and hoarseness to life-threatening conditions such as pneumonia, tracheal necrosis or rupture have been described in people and animals [12,13,14,15,16,17].

Many techniques for ETT cuff inflation have been described in the last few decades in human and veterinary patients. Commonly used techniques to assess cuff pressure include palpation of the pilot balloon (palpating the cuff’s pilot balloon while inflating, until the pressure felt is deemed adequate), assessing the minimal occlusive volume (inflation of the cuff until no audible leak is heard when applying positive pressure ventilation with a peak pressure of 20 cmH_2_O) or the minimal leak volume (after initial overinflation, slowly deflating the cuff just until a small leak is heard when applying positive pressure ventilation with a peak pressure of 30 cmH_2_O) and the use of syringe plunger recoil (inflating the cuff with a low resistance syringe and letting the plunger passively recoil) [8,9,18,19,20,21,22,23]. All of these techniques, even when used by experienced operators, rarely result in an optimal ETT cuff pressure [8,9,21,24]. Many authors therefore emphasise the necessity for direct measurement of the HVLP cuff pressure using a manometer [19,20,21,23,25,26,27,28,29,30,31].

Nowadays, a diverse range of cuff manometers are available [9,32]. Some common examples are aneroid manometers, such as the VBM cuff pressure gauge, the Posey Cufflator or special cuff inflation syringes with included pressure gauges, such as the Tru-Cuff and the AG-Cuffill syringes. Many veterinary facilities use manual techniques, such as those stated above, or they use repurposed aneroid manometers that are normally used for blood pressure measurement due to greater availability and lower cost. The use of repurposed manometers, however, is not recommended, as they are neither made to measure the low pressures required for cuff inflation, nor do they produce reliable readings [33].

With ease of use and cost being a major concern, especially for smaller veterinary facilities, the aim of this study was to find a cost-effective alternative to commercially available cuff manometers. Two types of disposable manometers, commercialized for use in respiratory resuscitation devices, were chosen considering their affordable price and portability. The purpose of this study was to test both of the disposable manometers for their usability, accuracy, precision and repeatability in ETT cuff inflation. The hypothesis was that both devices would be easy to adapt to use for ETT cuff inflation and show accurate and repeatable results.

## 2. Materials and Methods

**Figure 1:**
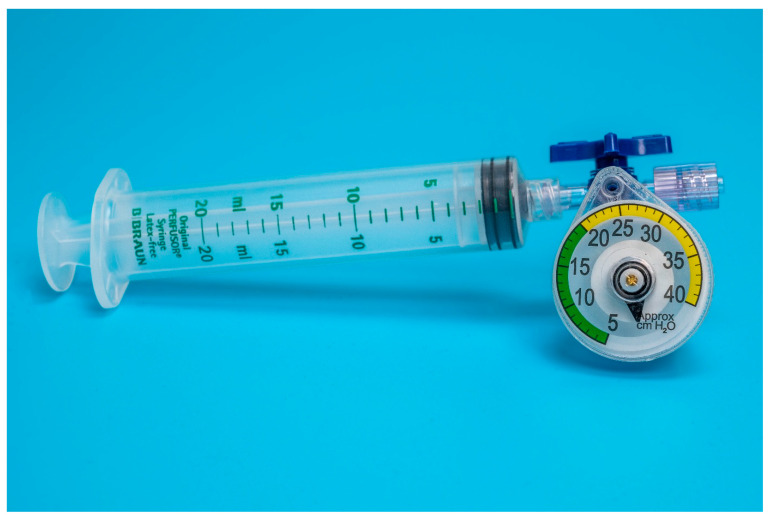
Setup of device A adapted for endotracheal tube cuff inflation.

**Figure 2:**
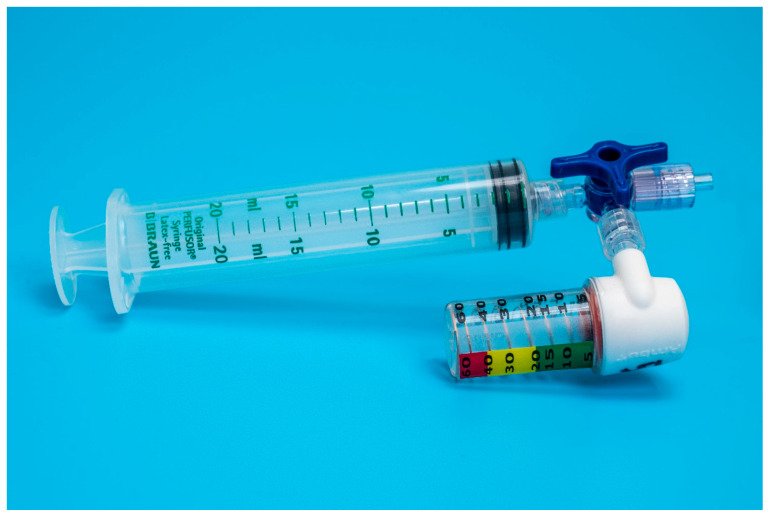
Setup of device B adapted for endotracheal tube cuff inflation.

Eighteen units of two commercially available disposable airways manometers–device A (Mercury Medical Single-Patient-Use Disposable Airway Pressure Manometer; Mercury Medical^®^, Clearwater, FL, USA; Figure 1) and device B (AMBU^®^ Disposable Pressure Manometer for single patient use; AMBU^®^ A/S, Ballerup, Denmark; Figure 2) were tested in a bench top model. Device A incorporates a spring-loaded membrane that deflects a pointer seated in a helicoid, while device B incorporates a spring-loaded piston that is directly deflected by changes in pressure. For device A, the manufacturer specifies an accuracy of ±5 cmH_2_O for pressures above 15 cmH_2_O. For device B, the specifications for accuracy are ±2 cmH_2_O for pressures up to 30 cmH_2_O.

### 2.1. Experimental Setup

**Figure 3:**
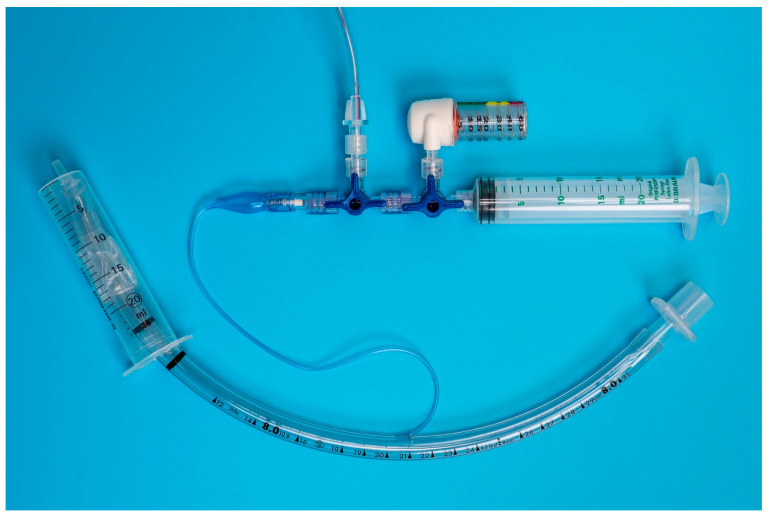
Benchtop setup: The endotracheal tube is placed in the model trachea (20 mL Syringe barrel) and connected via two 3-way stop cocks to the disposable manometer (device B), U-tube manometer (ridged extension line exiting the picture on the top) and syringe used for cuff inflation.

**Figure 4:**
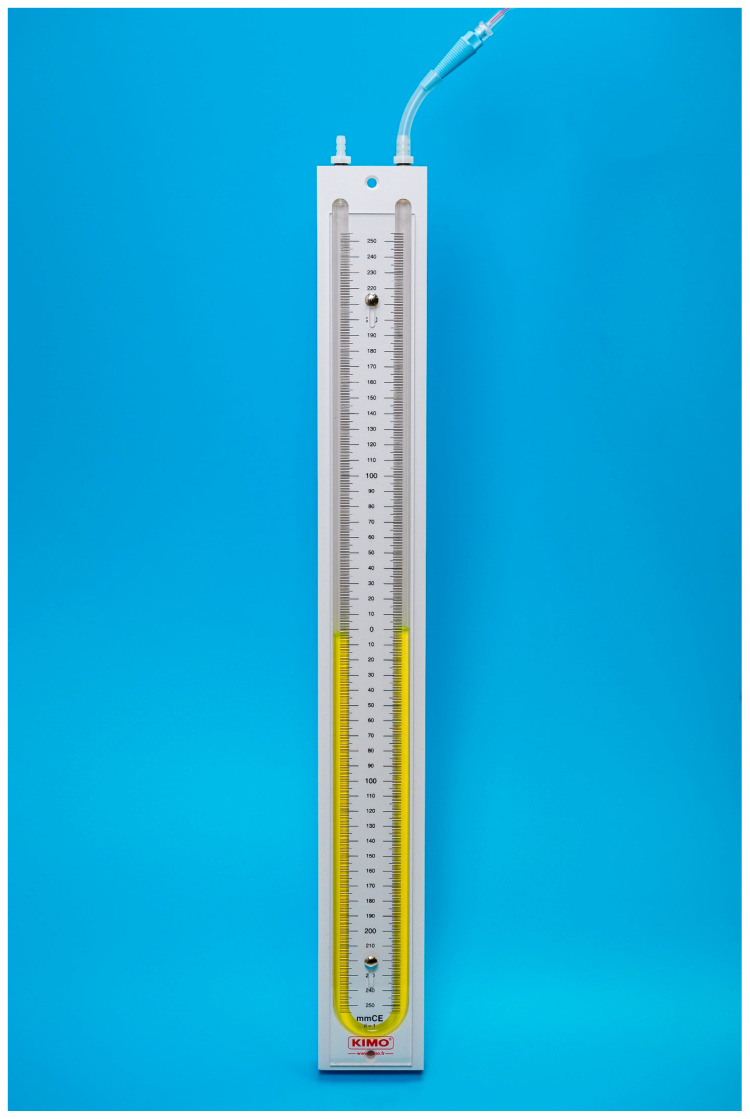
U-tube manometer used to measure true intra-cuff pressure. The U-tube manometer was connected via a ridged extension line to the rest of the benchtop setup.

Each unit was individually connected via two three-way stopcocks (Figure 1 and Figure 2) to the inflation valve of a new, size 8.0 I.D. polyvinyl chloride HVLP cuffed ETT (Shiley™Cuffed, Intermediate; Medtronic, Minneapolis, MN, USA). The same ETT was used for all the measurements. The cuff of the ETT was placed into the syringe barrel of a 20 mL syringe (B. Braun Injekt™ Syringes; B. Braun Melsungen AG, Melsungen, Germany), emulating a trachea (Figure 3). For device B, a male-to-male luer-lock to luer-slip adapter was used to allow for connection to the three-way tap. On the remaining two ports of the three-way stopcocks, a 20 mL luer-lock syringe (Original PERFUSOR^®^ Syringe Latex-free; B. Braun Melsungen AG, Melsungen, Germany), used for inflating the cuff, as well as a non-compliant extension line leading to a U-tube manometer with an accuracy of ±0.1 cmH_2_O (Vertical Liquid column manometer GF series VF1, Kimo Instruments, Montpon, France, Figure 4) were connected. The U-tube manometer was fixed to a vertical panel and checked for plumb with a water level, and the adjustable scale was zeroed to the meniscus of the liquid column. Prior to the experiment, the cuff of the ETT was checked for leaks by inflating it to a pressure of 50 cmH_2_O and checking for loss of pressure after ten minutes. Likewise, the system, excluding the disposable manometers, was tested for leaks by inflating to 50 cmH_2_O and checking for loss of pressure after 10 minutes.

### 2.2. Experimental Protocol

Each unit of the tested manometers was connected to the system as described above and three consecutive measurements were performed at target pressures of 20, 25 and 30 cmH_2_O, respectively. For each measurement, the system was overinflated by approximately 10 cmH_2_O to overcome the compliance of the system, and subsequently deflated to the target pressure, as indicated on the disposable manometers. For device A, the pressure was set according to the indicator on the dial. For device B, the pressure was set using the lower border of the O-ring on the approximate mid-point of the printed values of 20 and 30 for a pressure of 20 and 30 cmH_2_O, respectively. The lower border of the O-ring, on the mid-point of the scale between the printed values of 20 and 30, was used to set a pressure of 25 cmH_2_O.

Immediately after the target pressure was reached, one of the experimenters (VC) noted the deviation of the water column from the zero point. The system was then fully deflated before a new measurement was initiated. The person performing the inflation cycles (MEK) was blinded to the true pressure readings on the U-tube manometer.

### 2.3. Assessment of Usabilitly

The usability of each device was assessed subjectively by a single investigator (MEK) using the following criteria: ease of adaption of devices for ETT cuff inflation, intuitivity of use, bulkiness of the devices and ease of reading the devices’ pressure dials.

### 2.4. Statistical Analysis

For an initial descriptive evaluation, the measurements of the U-tube manometer pressure for the two devices were shown with boxplots at the three target pressures of 20, 25 and 30 cmH_2_O. For each device, the mean, standard deviation (SD) and the maximum deviation from the target pressure were calculated per unit and target pressure. Subsequently, for each device, the mean, SD, variance and maximum deviation from the target pressure were calculated for each target pressure. The overall deviation of the U-tube manometer pressure from the target pressure was separated into components using a linear mixed model with the unit as a random effect and the target pressure (levels: 20, 25 and 30 cmH_2_O) as a fixed effect. The average deviation from the target pressure was interpreted as the target pressure specific bias of the device (i.e., accuracy), the variance component associated with the unit as a measure of device precision, and the residual as a measure of repeatability. The linear model assumption of the equal variances among the levels was tested with Bartlett tests. The residuals were visually assessed for normality with Q–Q plots. Descriptive statistics was performed with Excel (Microsoft Excel for Mac, Microsoft Corporation, Redmond, DC, USA), while statistical models were calculated using the program R (R: A language and environment for statistical computing. R Foundation for Statistical Computing, Vienna, Austria).

## 3. Results

For each device, 18 units were tested with three measurements performed at three target pressures each. A total of 324 measurements were obtained.

### 3.1. Usability

Both devices were easy to adapt for ETT cuff inflation. Device A could be directly attached to a syringe and the pilot balloon of the ETT through a three-way stopcock (Figure 1). For device B, an additional piece, a male-to-male luer-lock to luer-slip adapter, was needed to connect the manometer to the three-way stopcock (Figure 2).

While being a bit bulkier than device B, device A was more intuitive to use with an easy-to-read graduated dial, indicating pressure increments of 1 cmH_2_O. This made it simple to set the pressure for any of the three tested target pressures. The markings on device B were not very precise, with increments of only 5 cmH_2_O at 5–20 cmH_2_O of pressure and increments of 10 cmH_2_O for up to 50 cmH_2_O of pressure. Furthermore, there were no specific marks on the manometer indicating at which position of the piston the target pressure was set. Nevertheless, reaching an approximate mid-point between the printed values of 20 and 30 cmH_2_O was still intuitive.

### 3.2. Measurements

For device A, the average measurements for the tested units ranged from 18.1–20.9, 22.1–24.7 and 26.8–30.0 cmH_2_O, with standard deviations of ±0.1–1.2, ±0.1–1.0 and ±0.1–0.7 cmH_2_O at target pressures of 20, 25 and 30 cmH_2_O, respectively. The median [Q1;Q3] were 19.8 [19.0;20.2], 23.8 [22.9;24.1] and 28.4 [28.1;28.9] cmH_2_O at 20, 25 and 30 cmH_2_O, respectively.

For device B, the averaged measurements for the tested units ranged from 18.3–20.2, 22.4–25.6 and 27.9–30.1 cmH_2_O, with standard deviations of ±0.1–0.5, ± 0.1–1.2 and ±0.1–0.9 cmH_2_O at target pressures of 20, 25 and 30 cmH_2_O, respectively. The median [Q1;Q3] were 19.3 [18.8;19.9], 24.5 [24.;24.8] and 29.4 [28.7;29.8] cmH_2_O at 20, 25 and 30 cmH_2_O, respectively. Two units of device B failed in meeting the manufacturer’s specifications (±2 cmH_2_O)—one unit at 25 and 30 cmH_2_O and one unit at 25 cmH_2_O target pressures, respectively.

Further descriptive statistics for device A and device B can be found in Table 1.

For device A, the linear regression showed a significant influence of the unit (*p* < 0.001) on the measurement, with a variance component of 0.5 (cmH_2_O)^2^ (or a standard deviation of 0.7 cmH_2_O) and a residual error of 0.2 (cmH_2_O)^2^ (or a residual standard error of 0.5 cmH_2_O). The former was interpreted as precision, the latter as repeatability. Depending on the level of the target pressure, device A showed a significant difference (*p* < 0.001) in bias; the respective bias was −0.4, −1.4, and −1.7 cmH_2_O for target pressures of 20, 25, and 30 cmH_2_O. A Bartlett test indicated no significant differences of the variances among the target pressures (*p* = 0.95).

**Figure 5:**
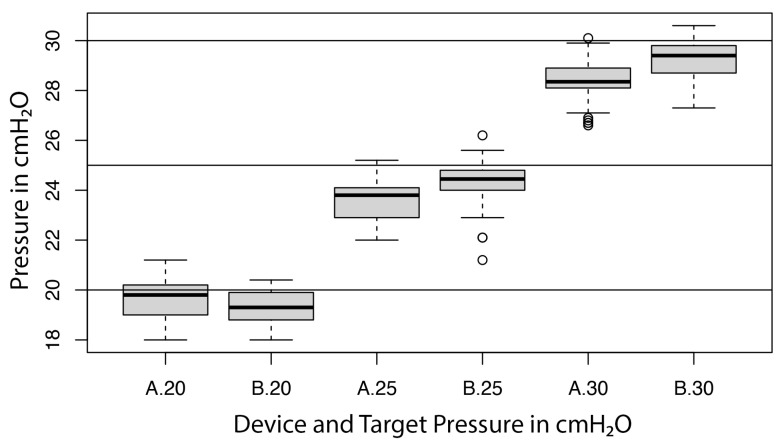
Boxplot illustrating the performance of both devices (A = device A; B = device B) at target pressures of 20, 25 and 30 cmH_2_O.

For device B, the linear regression showed a significant influence of the unit (*p* < 0.001) on the measurement, with a variance component of 0.4 (cmH_2_O)^2^ (or a standard deviation of 0.7 cmH_2_O) and a residual error of 0.2 (cmH_2_O)^2^ (or a residual standard error of 0.5 cmH_2_O). As above, the former was interpreted as precision, the latter as repeatability. The influence of the target pressure on the measurement was found not to be significant (*p* = 0.656); the respective bias of device B was −0.7, −0.7, and −0.8 cmH_2_O for target pressures of 20, 25, and 30 cmH_2_O. A Bartlett test indicated significant differences of variances among the three target pressures (*p* = 0.02). A visual inspection of the residuals indicated two possible outliers. The distribution of the measurements for device A and device B at each target pressure are represented in Figure 5.

## 4. Discussion

The aim of this study was to assess the usability, accuracy, precision and repeatability of two disposable airway pressure manometers at target pressures of 20, 25 and 30 cmH_2_O. The manufacturer’s specifications for accuracy at the tested target pressures were exceeded by all units except two of device B. Furthermore, both devices were easily adaptable for cuff inflation and easy to use.

While the inter-unit difference in pressure for both devices was significant at any target pressure (*p* < 0.001), the impact of this variability on clinical performance seems negligible, as standard deviations for both devices were well below 1 cmH_2_O. When looking further at the accuracy of the tested units for device A, all the units exceeded the specifications stated in the manufacturer’s data sheet (±5 cmH_2_O). Nevertheless, four units of device A would have failed the stricter specifications for device B (±2 cmH_2_O). For device B, two units did not meet the manufacturer’s specifications, with a maximum deviation of −2.6 cmH_2_O. This could have been due to the missing visual markings in device B for a pressure of 25 cmH_2_O. Considering a target pressure for cuff inflation between 20 and 30 cmH_2_O, the accuracy for both devices appear to be clinically sufficient.

Overestimation of the true pressure measured with the U-tube manometer was present in both devices. In device B, no significant change in bias could be found with an increase in the target pressure (*p* = 0.656). This was also evident by a very consistent mean deviation from the target pressure (bias) between −0.7 and −0.8 cmH_2_O. In device A, an influence of the target pressure on the bias was found (*p* < 0.001), resulting in an increased overestimation of the true pressure with an increase in the target pressure. The overestimation of the true pressures found in the study has little relevance in clinical conditions in which the aim to maintain the cuff pressure within the recommended range may be easily achieved by choosing 25 cmH_2_O or 30 cmH_2_O as target pressures.

With a variance component of 0.5 (cmH_2_O)^2^ (or a standard deviation of 0.7 cmH_2_O) and a variance component of 0.4 (cmH_2_O)^2^ (or a standard deviation of 0.7 cmH_2_O) for devices A and B, respectively, both devices showed clinically adequate levels of precision. In device B, a significant difference in the variances between the target pressures was found. The higher variance of device B at a target pressure of 25 cmH_2_O is most likely the result of the missing markings on the scale of the manometer for the specific target pressure, as previously mentioned. Therefore, a higher inter-measurement error can be expected at that target pressure in device B.

Both devices showed adequate levels of repeatability for ETT cuff inflation, with a residual error of 0.2 (cmH_2_O)^2^ (or a residual standard error of 0.5 cmH_2_O) and a residual error of 0.2 (cmH_2_O)^2^ (or a residual standard error of 0.5 cmH_2_O) for device A and B, respectively.

Most of the commercially available cuff manometers report an accuracy of ±2 cmH_2_O (VBM Cuff Manometer, Portex Posey Cufflator, AG Cuffill), similar to the devices tested in this study. Literature comparing commercial cuff manometers in accuracy and precision is somewhat sparse and often biased. In a study from 2004, Blanch compared four commercial cuff manometers in a benchtop model with a pressure range between 10 and 65 cmH_2_O. He reported a mean accuracy of the different devices between −0.9 and +0.7 cmH_2_O with a standard deviation of ±1.2–1.9 cmH_2_O [32]. Raft tested 27 manometers from three brands. Their mean ± SD deviation from the target pressures of 20, 27 and 30 cmH_2_O were 1.2 ± 1., 1.1 ± 1.1 and 1.2 ± 1.0 cmH_2_O, respectively [34]. Ramesh validated the AG Cuffill syringe in an in vitro and in vivo setting and reported a total mean bias ± precision of −1.9 ± 0.6 cmH_2_O over 300 measurements in three differently sized ETTs with a pressure range between 0 and 100 cmH_2_O [35]. Most of the above-mentioned studies report an increase in bias with an increase in the pressure, similar to our findings for device A. However, the upper pressure limits for the studies by Ramesh and Blanch were 65 and 100 cmH_2_O, respectively, potentially resulting in a larger bias compared to a pressure range between 20 and 30 cmH_2_O [32,35]. Blanch and Raft tested devices that were already used, which could potentially result in a loss of accuracy with age [32,34]. Ultimately, the majority of cuff pressure gauges available on the market show a variation of the inflation pressure. This variation is generally considered clinically insignificant and in line with our study results [34].

Several limitations of the present study need to be considered. First, the experimental setting cannot exactly reproduce in vivo conditions. A rigid syringe barrel used as a model trachea cannot perfectly replicate the conditions found in vivo. However, it is unlikely that this will have affected the measurements, as the study did only evaluate static and not dynamic changes in cuff pressure. Furthermore, only one size 8 mm I.D. HVLP ETT with an appropriately sized model trachea (20 mL syringe barrel) was used for all the measurements. A similar setup has been frequently used in comparable studies and represents an easily reproducible and economically viable model for ETT cuff inflation studies [36,37]. Only three measurements per unit at each target pressure were performed. Ideally, a higher number of measurements should have been obtained. However, three measurements were deemed adequate to gain a mean value for each unit at each target pressure. Furthermore, the experiment had to be conducted within one day to ensure similar environmental conditions and experimenter performance for all the measurements. Further research in vivo regarding the use of these manometers for ETT cuff pressure measurement is required in order to confirm the results. Second, due to practical and economic reasons, the number of units tested for each device were limited and belonged to the same production lot. The sample size was limited to 18 units of device A, therefore, the same number of units was chosen for device B to have an equal number of samples. Ideally, a larger number of units and from different batches should have been tested. Therefore, it is possible that different results may have been obtained if units from different batches were included in the study. Third, both devices tested are marketed for single use. Neither of the devices was tested for longevity and accuracy over time. It is therefore unclear how the devices function over an extended period of time, and it cannot be guaranteed that they will maintain the same level of accuracy as shown in this experiment. The authors anecdotally report the use of these devices for a four-month period at their institution. No noticeable loss of accuracy was experienced during clinical use and occasional comparison with commercial cuff manometers. Despite their recommended use as disposable units, it should be possible to re-use both devices, given that they are checked for accuracy on a regular basis against a reliable manometer. Similarly, checking commercially available manometers for accuracy on a regular basis has been suggested in the literature and by some manufacturers [34]. Lastly, while the usability of the devices was considered easy and intuitive by the experimenter, no large-scale user trials have been performed. The devices’ usability for ETT cuff inflation was only assessed by a single experimenter in this study. Despite other clinicians having a similar impression when using and handling the devices in a clinical setting, the findings remain subjective.

Both devices are very affordable, with a price approximately one tenth to one twentieth of that of commercial aneroid cuff manometers available on the market at the time of this study. Therefore, both devices can be seen as cost-effective and portable alternatives, allowing direct cuff pressure measurements in low-budget settings.

## 5. Conclusions

In conclusion, devices A and B may represent easy to use, cost-effective alternatives to more expensive devices for HVLP cuff pressure measurement. Both devices demonstrated clinically acceptable accuracy, precision and repeatability at target pressures between 20 and 30 cmH_2_O. Due to overestimation of the pressure, the authors suggest using the devices at target pressures between 25 and 30 cmH_2_O in order to maintain the ETT cuff pressure within the desired safe range. However, further research is warranted to confirm the results in in vivo settings and the reliability of the readings with prolonged use of the devices.

## Figures and Tables

**Table 1:** Descriptive statistics for device A and device B at target pressures of 20, 25 and 30 cmH_2_O.

	Device A			Device B		
Target Pressure (cmH_2_O)	20	25	30	20	25	30
Mean pressure (± SD)	19.6 (±0.7)	23.6 (±0.8)	28.3 (±0.8)	19.3 (±0.6)	24.3 (±0.9)	29.2 (±0.7)
Variance	0.5	0.6	0.6	0.4 *	0.8 *	0.4 *
Maximum deviation from target pressure	−1.9	−2.9	−3.2	−1.7	−2.6	−2.1

* The Bartlett test indicates significant differences between variances at the target pressures (*p* = 0.02).

## Data Availability

Data can be provided by the corresponding author on request.
